# Curcumin-zinc framework encapsulated microneedle patch for promoting hair growth

**DOI:** 10.7150/thno.84118

**Published:** 2023-06-26

**Authors:** Yating Yang, Pei Wang, Yan Gong, Ziyou Yu, Yuci Gan, Peizhe Li, Wei Liu, Xiansong Wang

**Affiliations:** Department of Plastic and Reconstructive Surgery, Shanghai Key Laboratory of Tissue Engineering, Shanghai Ninth People's Hospital, Shanghai Jiao Tong University School of Medicine, Shanghai 200011, China.

**Keywords:** zinc organic framework, curcumin, microneedles, hair loss, androgenic alopecia

## Abstract

Hair loss is a growing esthetic condition driven by complex mechanisms that has numerous psycho-social implications. Conventional drug applications usually focus on a single treatment target, and the penetration depth restricts the post-delivery effect.

**Method:** We fabricated a curcumin-zinc framework (ZnMOF) encapsulated gamma-polyglutamic acid (γ-PGA) microneedle patch (ZnMOF-MN) as a multifunctional biosafe transdermal drug delivery system. ZnMOF was characterized with the field emission scanning electron microscope (FE-SEM), dynamic light scattering (DLS), elemental mapping, and X-ray diffraction (XRD). The topographical and hygroscopic features of ZnMOF-MN were characterized with SEM. The *in vitro* ZnMOF release profile and the *in vivo* penetration of ZnMOF-MN were also evaluated. The anti-oxidant, anti-apoptosis, and antiandrogen effects of ZnMOF solution and ZnMOF-MN extract were studied on mouse dermal papilla cells (DPCs). Two animal models (in C57BL/6 mice), including androgenic alopecia (AGA) model and wound healing model, were used to identify the therapeutic effect of ZnMOF-MN on hair regrowth and wound healing *in vivo*. Hair follicles, surrounding vessels (CD31+), and proliferating cells (Ki67+) were evaluated by histological staining.

**Results:** ZnMOF crystals were cone-shaped nanoparticles with a size distribution of 424.9 ± 59.01 nm. ZnMOF-MN patch can create temporary holes in the skin to directly and evenly deliver bioactive ZnMOF particles to the targeted depth and achieve a steady and sustained release of Zn^2+^ and curcumin. *In vitro*, ZnMOF significantly improved the viability of DPCs against the excess reactive oxygen species (ROS) and inhibited the apoptosis induced by zinc deficiency. In addition, it also reversed the inhibitory effects of dihydrotestosterone (DHT) infiltration. Moreover, the ZnMOF-MN treatment has been proved to accelerate wound healing and increase hair follicles in wound healing models, and improved the hair regrowth in AGA animal models. Enhanced capillary density and cell proliferation observed in the CD31+ and Ki67+ staining of ZnMOF-MN group in both animal models also suggested that ZnMOF can facilitate angiogenesis and promote cell proliferation in the skin, respectively.

**Conclusion:** The ZnMOF-MN treatment is a comprehensive solution with excellent therapeutic efficacy and patient-friendly features for promoting hair growth under various clinical conditions.

## Introduction

Hair loss is a growing esthetic condition with various psycho-social implications. According to a recent report, up to 85% males and 40% females are bothered with hair conditions 1. Hair follicles undergo periodic changes, including catagen (regression phase), telogen (resting phase), and anagen (growth phase) phases, to produce new hairs. A dysregulated local microenvironment can negatively affect the hair cycle, which is a major and common cause of hair loss of various types. Oxidative stress, which is prevalent in the pathological microenvironment of alopecia, is caused by the presence of excess reactive oxygen species (ROS) 2. The concentration of malondialdehyde, which represents oxidative stress, is proportional to the course and severity of the disease 3, and increased levels of it have been detected in both plasma samples and scalp biopsies of patients with androgenetic alopecia (AGA) 3,4. Nutrient conditions are also considerable factors affecting hair growth. As previously reported, insufficient blood perfusion of skin may decrease the local density of cytokines and nutrients, and hinder the telogen-to-anagen transition 5. In clinical, local and systemic zinc (Zn, Zn^2+^) deficiencies are closely related to the severity and chronicity of the disease 6,7, and are commonly observed in hair loss due to various causes, including diffuse alopecia 8, telogen effluvium 7,9, and alopecia areata 6,7. In addition, hormones play significant roles in the mechanism of AGA 10, which induce the anagen-to-catagen transition by down-regulating the Wnt/β-catenin pathway 11,12, promoting dermal papilla cell (DPC) apoptosis via the prostaglandin D2 pathway 13, and stimulating the local ROS enrichment 3. Traditional chemicals (such as minoxidil) and AGA animal models have often been used in previous studies 14-16. Unfortunately, alopecia caused by other factors such as nutrient deficiency or injury is neglected.

Zn is present in numerous enzymes and protein domains, making it essential for growth and development, and plays a key role in the regulation of apoptosis and inflammation 17,18. Hair is the third most zinc-abundant tissue in the body 19. Zinc deficiency has a broad negative impact on keratinocytes, fibroblasts, vasculature, and the general health of hair 20-23. Clinical studies have revealed that zinc deficiency is prevalent in different types of alopecia 6-9, and zinc supplementation is strongly recommended in patients with hair problems 7. Curcumin, derived from Curcuma longa L., has been used as herbal medicine in a wide range of fields 24, including in treating skin disorders 25, neurological diseases 26, and cancers 27. As a natural ROS scavenger, curcumin has been proved to have anti-oxidant and anti-inflammatory effects on skin aging, dermatitis, and wound healing 25. In addition, curcumin analogues have been reported to have anti-androgen effects by inhibiting androgen receptor activation and steroid 5-alpha reductase 28,29. Moreover, curcumin is well tolerated with a maximum daily intake of 3 mg kg^-1^ or 12 g day^-1^ 30, which renders it a biosafe drug with great potential. Unfortunately, curcumin has poor bioaccessibility, and its low water solubility, low stability, and extensive first-pass metabolism considerably limit its further application 8,25. Therefore, a stable topical transdermal drug delivery system that can steadily deliver Zn^2+^ and curcumin directly to the dermal layer is required to maximize their biological effects.

Featured with tunable composition and enhanced biocompatibility, metal-organic frameworks (MOFs) are crystalline porous materials self-assembled by metal ions and organic ligands under specific circumstances 31. Previous studies on Mg-MOFs and Cu-MOFs in diabetic wound healing models have proved that the MOF structure is reliable in controlling metal release and maintaining the bioactivity of organic substances under complicated microenvironment 32,33. Thus, MOFs are promising nanoplatforms for controlled drug delivery.

Soluble polymer microneedle (MN) patches are powerful transdermal drug delivery systems 34,35. MNs with nanoparticle-encapsulated tips can penetrate the epidermis and transport drugs to certain skin depth 36,37. Moreover, penetration with less than 750 μm depth can barely cause bleeding or pain 38. The utilization of MN patches in treating alopecia was first introduced in 2009 39, and has boomed since 2020. Therefore, MN administration could be an effective, safe, and patient-friendly method of transdermal drug delivery for treating hair loss.

Herein, we introduced curcumin and zinc ions into MOFs (ZnMOFs), and encapsulated them with gama-polyglutamic acid (γ-PGA) to make soluble ZnMOF-MN patches for promoting hair growth under various conditions. γ-PGA hydrogel is often used as the base material for MN fabrication as it can provide sufficient mechanical strength after drying and have excellent biodegradability and biological activity 40,41. Meanwhile, ZnMOFs can protect DPCs from apoptosis induced by zinc deficiency and oxidative stress. Moreover, ZnMOFs also promoted the expression of genes related to DPC proliferation (PCNA) and Wnt/β-catenin signaling activation (CCND1, AMER3, LEF1, DDK1) 42, and down-regulated genes related to apoptosis (Caspase-3, Caspase-9) and androgen metabolism (HSD17B2, SRD5A1). As a result, accelerated wound healing and promoted hair regrowth in traumatic models, exhibiting a broad-spectrum therapeutic effect against the complicated mechanisms in scalp with hair loss problems, including zinc deficiency, ROS infiltration, and poor cell growth were testified both *in vivo* and *in vitro*. Thus, ZnMOF-MNs, which have broad applications in promoting hair growth under various conditions, can act as effective therapeutics for hair loss problems.

## Results and Discussion

### Synthesis and characterization of ZnMOF

**Figure 1:**
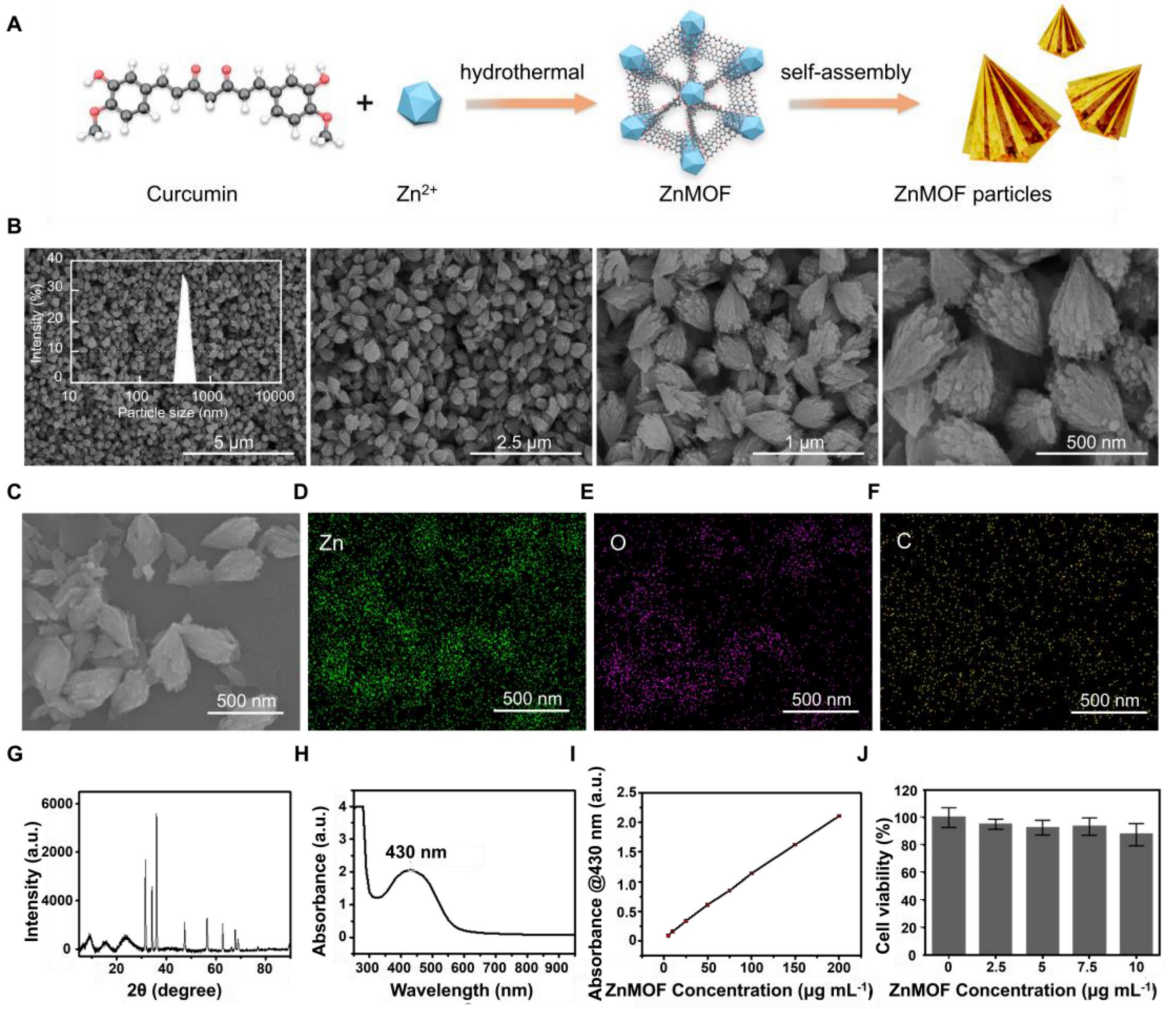
** Synthesis and characterization of ZnMOF. A.** Schematic illustration of the synthesis of ZnMOF microsphere. **B.** FE-SEM image and size distribution of ZnMOF. **C-F.** SEM image and elemental mapping of ZnMOF. **G.** XRD spectra of ZnMOF powders.** H.** Absorption spectrum of ZnMOF. **I.** Absorption intensity at 430 nm with different concentrations of ZnMOF. Error bars are shown in red. **J.** Cell viability of DPCs treated with various concentrations of ZnMOF (0, 2.5, 5, 7.5, and 10 μg mL^-1^) for 48 h. No statistical significance was observed among all groups.

ZnMOF was synthesized by the reaction between curcumin and zinc ions (Zn^2+^) in alkaline water (pH = 12), which then self-assembled into cone-shaped nanoparticles (Figure 1A-B). Field emission scanning electron microscope (FE-SEM) image and dynamic light scattering (DLS) analysis indicated that ZnMOF nanoparticles have a size distribution of 424.9 ± 59.01 nm (Figure 1B). Elemental mapping showed homogeneous distributions of carbon (C), oxygen (O), and Zn within the ZnMOF (Figure 1C-F), and the weight percentage of Zn was 14.99%. The X-ray diffraction (XRD) was used to analyze the crystal structure of the ZnMOF powder. Figure 1G showed the XRD patterns of ZnMOF particles, the standard diffraction peaks of which are consistent with the crystalline nature of zinc crystals 43, suggesting the successful synthesis of ZnMOF with an integral crystal structure. Besides, the absorption profile of the ZnMOF solution also shows a characteristic peak at 430 nm (Figure 1H-I), consistent with that of curcumin 44. As Figure 1J indicated, no obvious cytotoxicity of ZnMOFs to mouse DPCs was observed at concentrations up to 10 μg mL^-1^. Thus, we successfully fabricated a stable and biocompatible metal-organic framework consisting of Zn and curcumin.

### Synthesis and characterization of ZnMOF-MN

**Figure 2:**
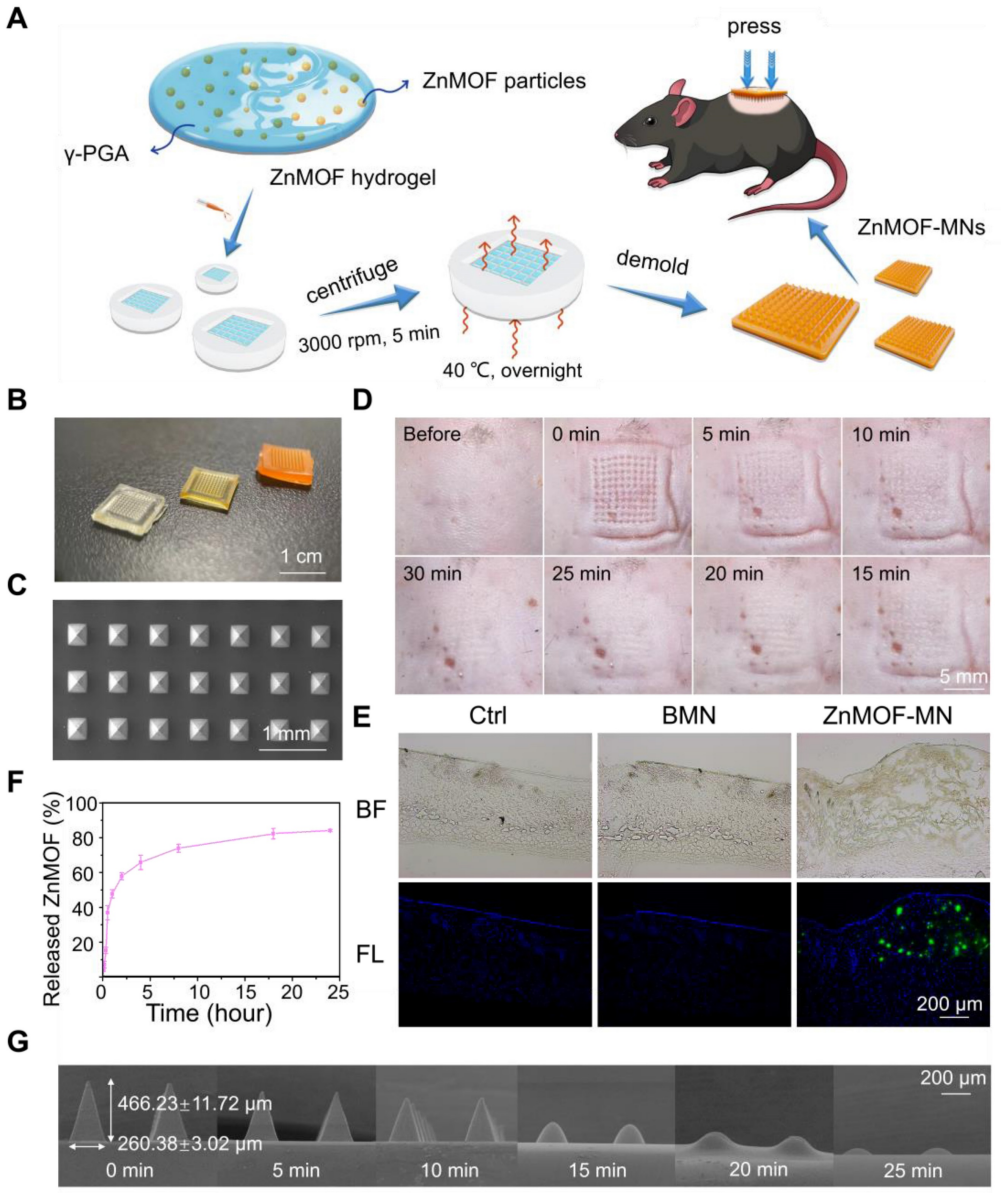
** Synthesis and characterization of ZnMOF-MN. A.** Schematic illustration of the synthesis process of ZnMOF-MNs. **B.** Photographic image of MN patches (from left to right: BMN, ZnMOF-MN (0.1 mg mL^-1^), and ZnMOF-MN (1 mg mL^-1^)). **C.** Top-down SEM image of a patch of ZnMOF-MN. **D.** Recovery procedure after ZnMOF-MN penetration in the back skin of a C57BL/6 mouse. **E.** Brightfield (BF) and fluorescence (FL) microscopic images of untreated C57BL/6 mouse skin (the control group, Ctrl) and skin penetrated with BMN or ZnMOF-MN. ZnMOF showed green fluorescence signals. **F.** Release profile of ZnMOF from a ZnMOF-MN in PBS within 24 h. **G.** SEM images of ZnMOF-MN tips after moisture absorption at different time points (75% humidity, 25 °C). The labels revealed the detailed dimensions of the MN tips: a base diameter of 260.38 ± 3.02 μm and a height of 466.23 ± 11.72 μm.

Soluble ZnMOF-MN was made of γ-PGA which has been widely used owing to its excellent biodegradability, biological activity, and proper mechanical strength after drying 45. MNs were fabricated using commercial polydimethylsiloxane (PDMS) MN molds according to the procedures presented in Figure 2A. Equal volume of ddH_2_O was used to replace the ZnMOF solution when fabricating blank MNs (BMN). As shown in Figure 2B, the orange-yellow color became more prominent with increase in ZnMOF concentration, indicating the stable existence of curcumin during production. No obvious cytotoxicity to mouse DPCs was observed when treated with the BMN or ZnMOF-MN extracts at the experimental concentrations (Figure S1). The top-down SEM images were recorded for presenting the topographic characteristics of ZnMOF-MN, which showed that the pyramid-shaped tips were orderly aligned (10 × 10 array) in MN patches (Figure 2C) with average height of 466.23 ± 11.72 μm (Figure 2G). *In vivo* studies showed that these pyramidal tips were sharp enough to penetrate the skin and create temporary perforations which recovered within 30 min without bleeding (Figure 2D). No obvious penetration-induced skin damage or inflammation was observed in 24 h (Figure S2). The penetrated skin was collected for making 8-μm-thick transverse cryosections immediately after the last photograph was captured. The transdermal delivery of ZnMOF was further confirmed by detecting fluorescence signals of curcumin (excitation at 423 nm; emission at 527 nm) beneath the skin (at ~400 μm, Figure 2E) 46. To measure the ZnMOF release profile in an aqueous environment, MNs were immersed in phosphate-buffered saline (PBS) and the absorbance of the supernatant at 430 nm was monitored over time. The release rate of ZnMOF was 57.86% at 2 h and 84.14% at 24 h, as determined by evaluating curcumin release (absorbance at 430 nm, Figure 2F) and 49.74% at 2 h and 87.67% at 24 h by evaluating the Zn^2+^ release (Figure S3). As for the hygroscopicity evaluation, ZnMOF-MN patches were placed inside a sealed tank with a humidity maintained at 75% (at 25 °C). The morphology of the MN tips was recorded every 5 min, and 90% of the tips were dissolved within 25 min (Figure 2G). Thus, soluble ZnMOF-MNs proved to be a stable and effective platform for drug release and transdermal delivery.

### *In vitro* anti-oxidant, anti-apoptosis, and anti-androgen capacities of ZnMOF

**Figure 3:**
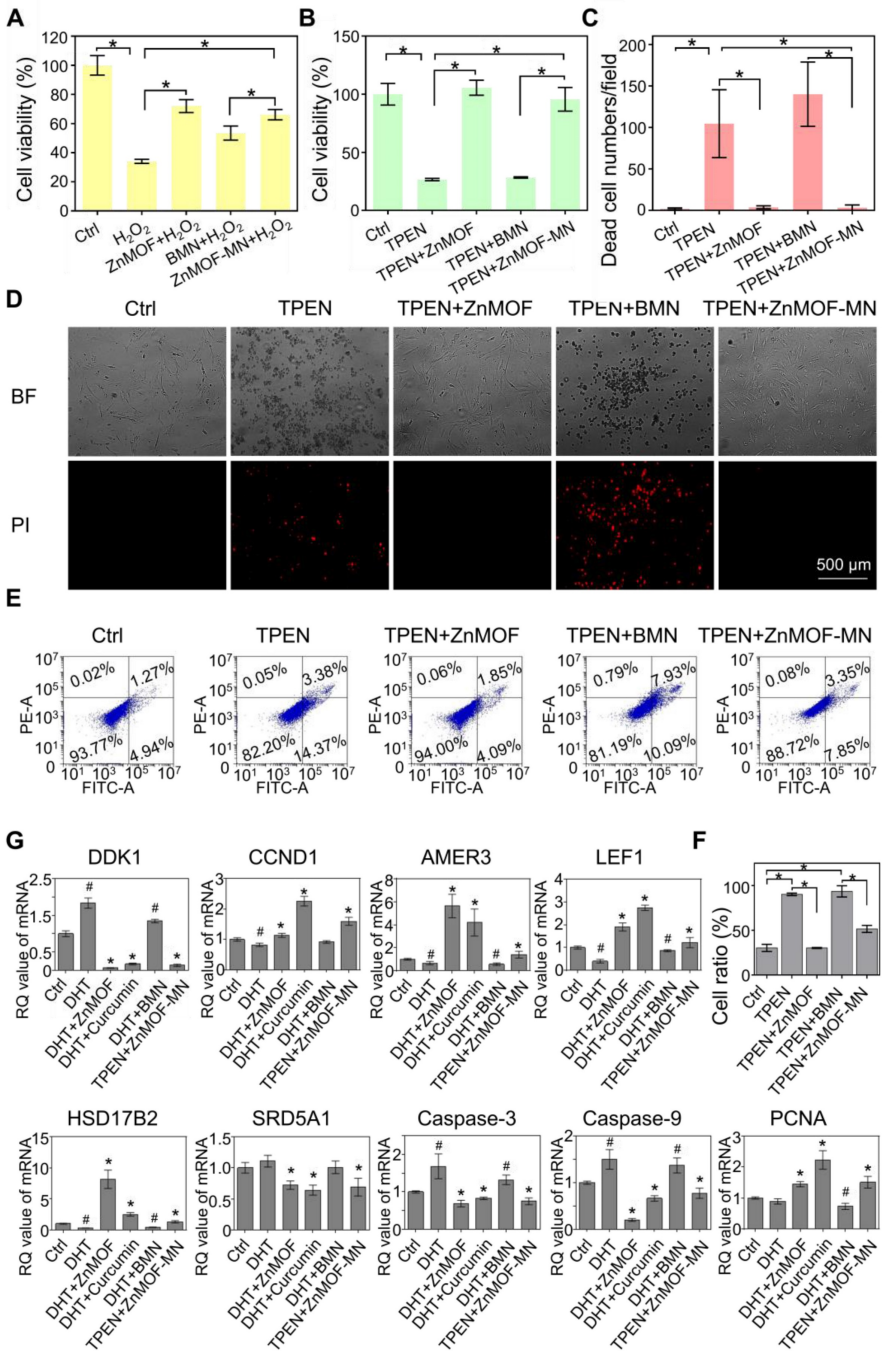
***In vitro* anti-oxidant, anti-apoptosis, and anti-androgen capacities of ZnMOF-MN. A.** Cell viability of DPCs treated with 5 μg mL^-1^ ZnMOF solution, BMN extract, or ZnMOF-MN extract (~6.46 μg mL^-1^ ZnMOF) for 24 h and then incubated with 3 mM H_2_O_2_ for 30 min.** B.** Cell viability of DPCs treated with 5 μM TPEN and 5 μg mL^-1^ ZnMOF solution, BMN extract, or ZnMOF-MN extract for 24 h. **C-D.** Representative images and quantification of dead DPCs stained with PI (red). Cell morphological changes in the bright field (BF) also reflect the cell condition. **E-F.** Representative images and ratio of apoptotic cells measured by flow cytometer. **G.** RT-qPCR analysis of DDK1, CCND1, AMER3, LEF1, HSD17B2, SRD5A1, Caspase-3, and Caspase-9 in DPCs treated with 100 μM DHT and 20 μM curcumin, 2.5 μg mL^-1^ ZnMOF, BMN extract, or ZnMOF-MN extract (~3.23 μg mL^-1^ ZnMOF) for 24 h. RQ values (relative quantitative value to the internal reference) of DPCs treated with 100 μM DHT and BMN extract were compared with the control group (Ctrl, 0.1% DMSO) while that of DPCs treated with 20 μM curcumin, 2.5 μg mL^-1^ ZnMOF, and ZnMOF-MN extract were compared with the DHT group. n=3; *p < 0.05 vs. Ctrl in **A-C**; ^#^p < 0.05 vs. Ctrl, *p < 0.05 vs. DHT in **G**.

As previously described, oxidative stress, zinc deficiency, and androgenic disorders are the prevailing risk factors for hair loss. Oxidative stress is defined as an imbalance between the production of free radicals (ROS) and antioxidant defenses 47. In the scalp, oxidative stress induced by various causes (such as aging, hormonal fluctuations, and traumatic events) is connected to hair cycle interruption, greying and hair fiber impairment 48. Curcumin is a potent antioxidant substance and has been used to scavenge excess ROS in diabetic conditions and wounded skin 48, 49. To confirm the antioxidative effect of ZnMOF and ZnMOF-MN, DPCs were pretreated with 5 μg mL^-1^ ZnMOF solution, BMN extract, or ZnMOF-MN extract for 24 h and then incubated with H_2_O_2_ for 30 min to simulate the excess ROS stress. The CCK-8 assay was used to evaluate cell viability. Both the ZnMOF solution and ZnMOF-MN extract showed potent antioxidant capacity, as they considerably reversed the decreased cell viability (Figure 3A). Moreover, ZnMOF showed superior anti-oxidant effect compared to curcumin or curcumin combined with Zn ions under similar concentrations (Figure S4), indicating the improved capacity of ZnMOF and ZnMOF-MN for anti-oxidation.

Zinc deficiency is a common cause of cell apoptosis during hair loss 14,15. To mimic the zinc deficient microenvironment, 5 μM TPEN was used to chelate Zn ions in the culture medium and induce cell apoptosis, as indicated by the decreased cell viability in the TPEN and BMN groups in the CCK-8 assay (Figure 3B). Furthermore, 24 h treatment with ZnMOF solution (5 μg mL^-1^) and ZnMOF-MN extract (~6.46 μg mL^-1^ ZnMOF) considerably restored the reduction in cell viability after TPEN incubation (Figure 3B). Propidium iodide (PI) can cross the dead cell membranes and bind to nucleic acids; therefore, it was used in this study to mark dead cells under zinc deficiency. As shown in Figure 3D, TPEN incubation induced prominent cell apoptosis, characterized by morphological changes (cell detachment and volume reduction) and PI staining. Quantification of PI staining positive cells indicated that the number of dead DPCs significantly decreased from ~105 cells per field in the TPEN group to ~4 cells per field in ZnMOF solution group and to ~3 cells per field in the ZnMOF extract group, which was different from that in the control group (~2 cells per field) under zinc deficient conditions (Figure 3C). Furthermore, flow-cytometric analysis revealed that the increase in total apoptosis rate (the sum of cell percentages in the UR and LR quadrants) triggered by TPEN incubation was compromised by the ZnMOF solution or ZnMOF-MN extract treatment (Figure 3E-F). Therefore, ZnMOF and ZnMOF-MN can reverse the biological effects of zinc deficiency by acting as effective topical Zn supplementation.

Dihydrotestosterone (DHT) infiltration is another inducer of poor hair follicle status, particularly in patients with AGA 11,50. As an active form of testosterone, DHT stresses DPCs and incurs chronic inflammation in hair follicles, leading to cell apoptosis and hair cycle recession 50. In this study, DHT incubation was used to simulate the AGA microenvironment, and transcriptional changes in DPCs were evaluated by reversing transcription quantitative polymerase chain reaction (RT-qPCR) to analyze the effects of 2.5 μg mL^-1^ ZnMOF solution and ZnMOF-MN extract (~3.23 μg mL^-1^ ZnMOF) on DHT-induced alterations. As shown in Figure 3G, 24 h incubation with DHT significantly up-regulated apoptosis-related genes, including Caspase-3 and Caspase-9. Treatment with curcumin (the positive control, 20 μM), ZnMOF solution, and ZnMOF-MN extract alleviated these negative effects and increased the expression of PCNA (indicating active cell proliferation activities) in DPCs. Moreover, the down-regulated Wnt/β-catenin signaling pathway is also responsible for the hair growth delay in AGA 11,12. To confirm the effect of ZnMOF on DHT-induced Wnt/β-catenin inactivation, we evaluated the expression of related genes in DPCs after DHT incubation through RT-qPCR. CCND1 is a Wnt target gene, whereas LEF1 and AMER3 are critical mediators of the Wnt/β-catenin signaling pathway. As shown in Figure 3G, the expression of genes involved in Wnt/β-catenin signaling activation (CCND1, LEF1, and AMER3) increased significantly, whereas those of the Wnt/β-catenin antagonist (DDK1) decreased significantly in DPCs exposed to curcumin, ZnMOF solution, or ZnMOF-MN extract (Figure 3G) 42,51. Collectively, ZnMOF and ZnMOF-MN were proven to offset the negative effects of DHT and restore the Wnt/β-catenin signaling pathway activation in DPCs in the AGA microenvironment.

Finally, curcumin has also been reported to exert potent inhibitory effects against testosterone production and androgen activation (testosterone-to-DHT transition) by modulating AKR1C2 and HSD3B2 expression 25,52, and by acting as a 5-α reductase inhibitors 26. Similarly, in this study we observed a significant decrease in HSD17B2 and SRD5A1 expression in DPCs treated with curcumin, ZnMOF solution, or ZnMOF-MN extract (Figure 3G), suggesting that ZnMOF-MN may exert a healing effect against the AGA microenvironment by reducing androgen production and activation. In conclusion, ZnMOF and ZnMOF-MN exerted therapeutic effect against DHT-induced cell apoptosis and Wnt/β-catenin inactivation and reduced the expression of androgen production-related genes at the transcriptional level.

### *In vivo* hair growth evaluation and histological study in C57BL/6 mice of wound healing models

**Figure 4:**
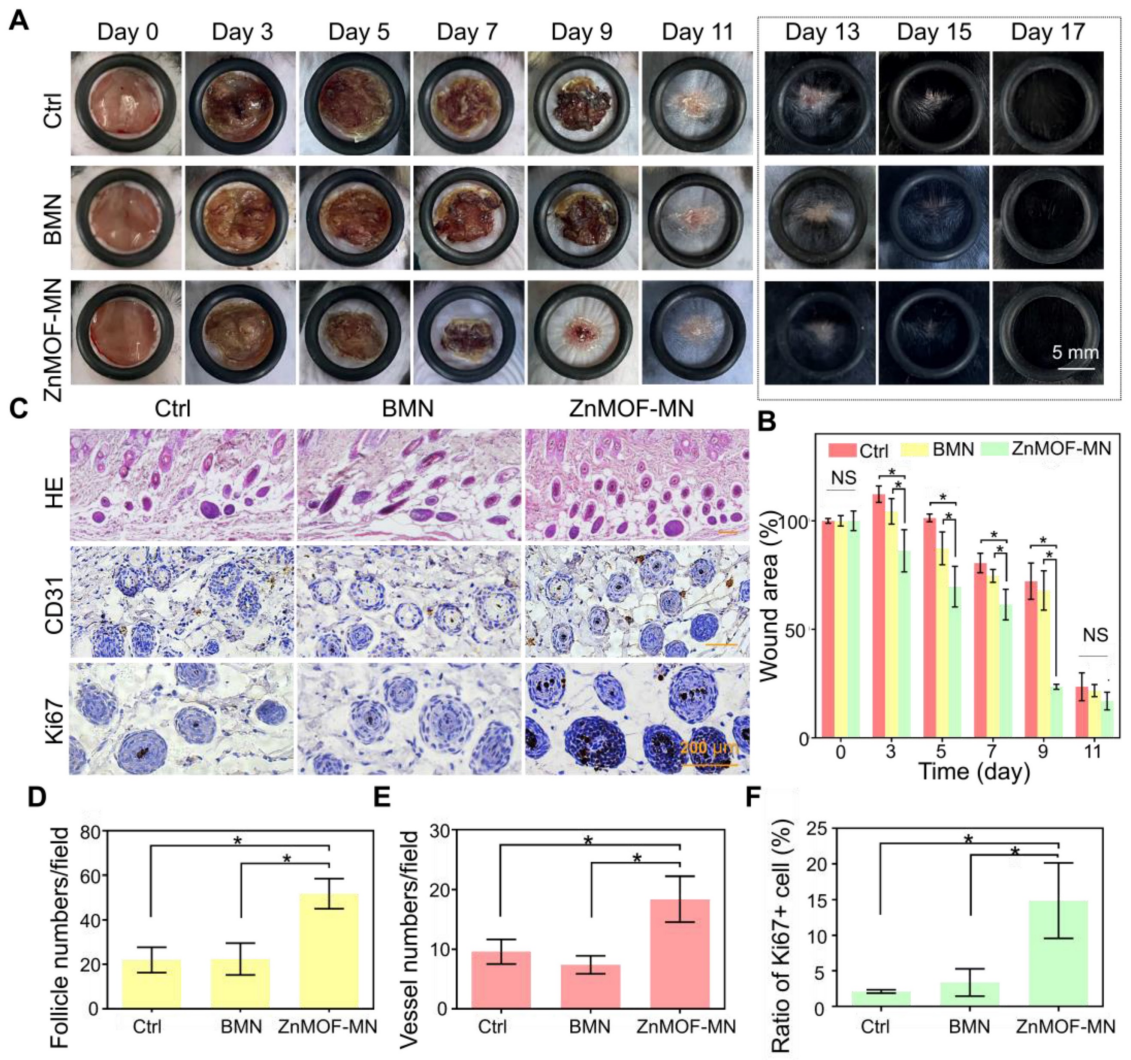
***In vivo* hair growth evaluation and histological study in C57BL/6 mice of wound healing models. A.** Representative photographic images of the wounded skin after different treatments on day 0, 3, 5, 7, 9, 11, 13, 15, and 17, respectively. The control group (Ctrl) was mice without treatment. **B.** Quantification of the wound area rate in each group on day 0, 3, 5, 7, 9, and 11. **C.** Images showing H & E staining (upper row), anti-CD31 immunohistochemistry staining (middle row), and anti-Ki67 immunohistochemistry staining (lower row) in different groups on day 17 (hair regrowth). **D.** Quantification of follicle number per field using H & E staining. **E.** Quantification of capillary density using anti-CD31 staining. **F.** Quantification of proliferating cell rate using anti-Ki67 staining. n = 3, *p < 0.05, NS refers to no significance.

Skin injury, particularly dermal layer damage, is a common cause of hair loss. To evaluate the healing effect of ZnMOF-MN under skin damage-induced hair loss conditions, a mouse model was established by creating round (~9 mm) full-thickness defect wounds on the backs of C57BL/6 mice (male, 6-8 weeks old). Mice in the control group (Ctrl) were not treated, whereas those in the BMN and ZnMOF-MN groups were treated with BMN and ZnMOF-MN, respectively. Photographs of mice in each group on day 0, 3, 5, 7, 9, 11, 13, 15, and 17 were recorded for further analysis (Figure 4A and Figure S5). As shown in Figure 4B, the wound area contracted faster in the ZnMOF-MN group before decrustation (day 11) than in the control group. The average ratios of wound area in the control, BMN, and ZnMOF-MN groups on day 9 were 72.16%, 67.93%, and 23.55%, respectively. Accelerated wound closure was also confirmed by the increased cell migration observed in DPCs treated with the ZnMOF-MN extract (Figure S6). Moreover, expression of M1 and M2 phenotype markers in RAW264.7 cell lines (M0) showed that ZnMOF-MN extract can reduce inflammation by down-regulating the proinflammatory M1 polarization (indicated by decrease in M1 marker expression, including IL-1β, iNOS, and TNF-α) and up-regulating the anti-inflammatory M2 polarization (indicated by increase in M2 marker expression, including IL-10, CD206, and TGF-β) *in vitro* (Figure S7), which may also contribute to the accelerated wound healing process. On day 17, the wounds in all groups were covered with regenerated hair, but hematoxylin and eosin (H & E) staining of skin samples revealed that the follicle number of the ZnMOF-MN group was double that of the control group and the BMN groups (Figure 4C-D). Immunohistochemistry staining for CD31 (Figure 4C) revealed denser capillary distribution surrounding the hair follicles in the ZnMOF-MN group (Figure 4E). Similarly, the ratio of Ki67 positive cells in the ZnMOF-MN group was almost five times higher than those in the other two groups (Figure 4C-F). These results suggested that ZnMOF-MN promoted hair regrowth during the wound healing process by accelerating wound closure, modulating the microvasculature, and stimulating cell proliferation.

### *In vivo* hair growth evaluation and histological study in C57BL/6 mice of AGA models

**Figure 5:**
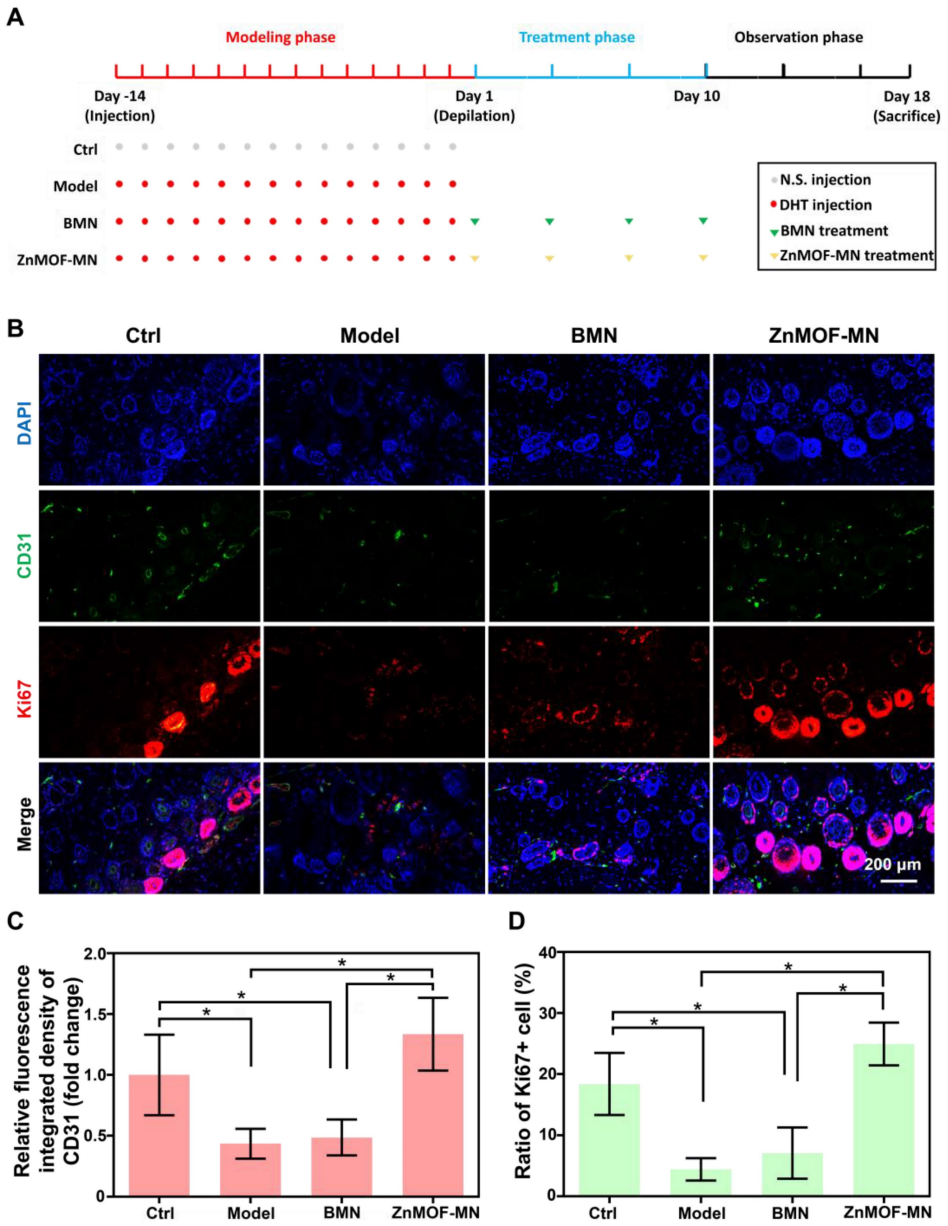
** Model establishment and histological study. A.** Schematic illustration of the AA model established in C57BL/6 mice. **B.** representative fluorescence images of anti-CD31 and anti-Ki67 staining in different groups.** C.** Quantification of capillary density using anti-CD31 staining. **D.** Quantification of proliferating cell rate by anti-Ki67 staining. n = 3, *p < 0.05. Ctrl for the control group.

**Figure 6:**
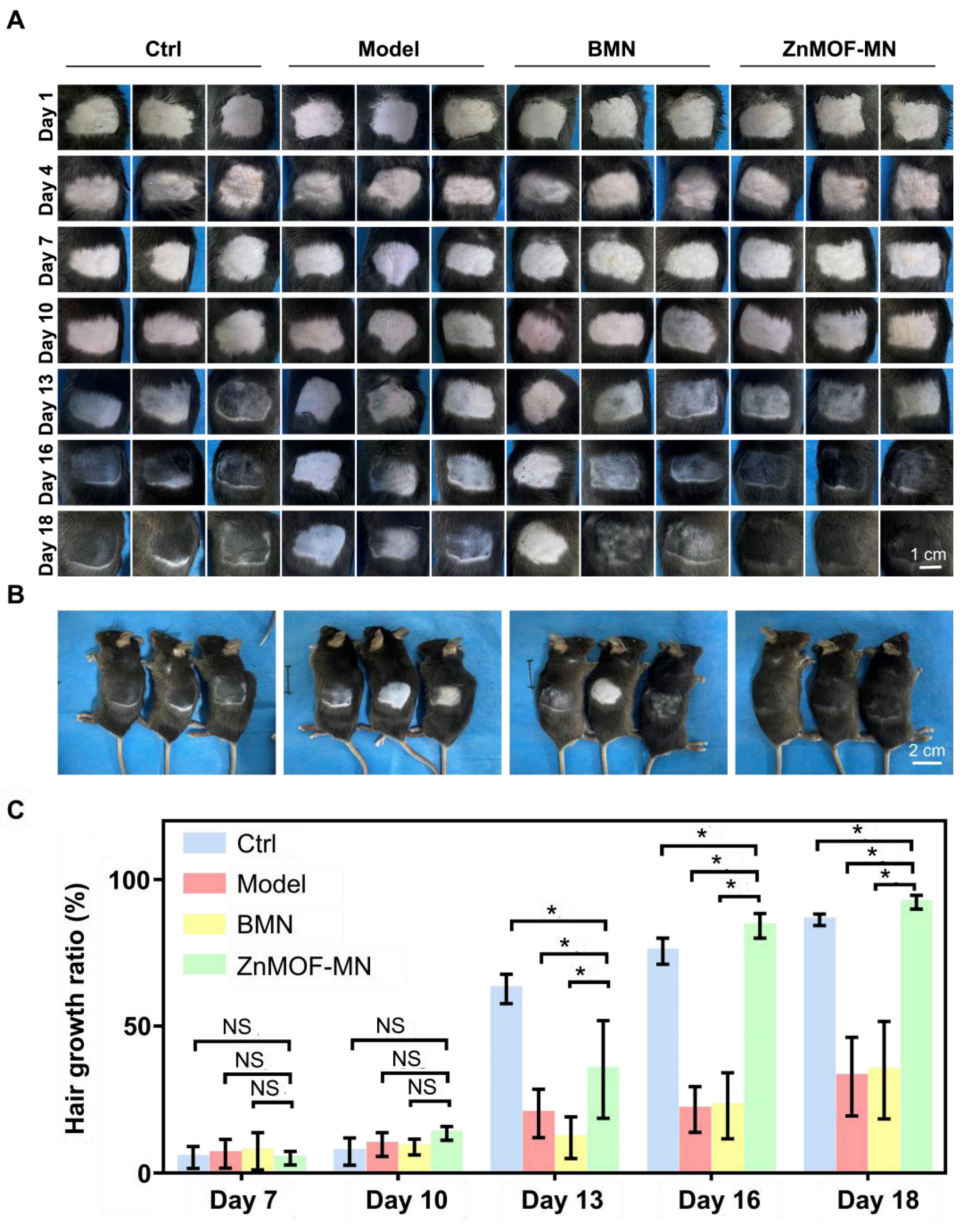
***In vivo*** **hair growth evaluation in C57BL/6 mice of androgenic alopecia models. A.** Representative images of the hair regrowth condition in different groups on day 1, 4, 7, 10, 13, 16, and 18 after depilation. **B.** Gross images of hair regrowth in mice of different groups on day 18. **C.** Quantification of the hair growth rate in each group on days 7, 10, 13, 16, and 18. n = 5, *p < 0.05, NS refers to no significance. Ctrl for the control group.

As the most prevalent type of alopecia, AGA is closely associated with internal abnormalities such as hormonal fluctuations and immune disorders. An AGA animal model was established according to a published study with minor adjustments, to confirm the therapeutic effect of ZnMOF-MN in the AGA microenvironment 53. As shown in Figure 5A, male C57BL/6 mice (6-8 weeks old) were subcutaneously injected 2 mg kg^-1^ DHT for 14 days. Mice treated with an equal volume of normal saline (N.S.) were used as the control group and depilated together with others on day 1. In the experimental groups, mice were treated with BMNs or ZnMOF-MNs on bare skin on day 1, 4, 7, and 10. No intervention was performed for mice in the Model group. As for mice in the Model group. Fluorescence images of anti-CD31 stained skin samples on day 18 also revealed that the impairment of capillary density due to DHT treatment was compromised in the ZnMOF-MN group (Figure 5B-C). Similarly, the Ki67-positive ratio (representing proliferating DPCs) that had decreased in the Model group and BMN groups was enhanced after ZnMOF-MN treatment (Figure 5B-D). Images of the depilated skin were recorded on day 1, 4, 7, 10, 13, 16, and 18 to evaluate hair regrowth (Figure 6A and Figure S8). On day 18, hair regrowth of mice in the Model and BMN groups were retarded (Figure 6A-B). In comparison, mice treated with ZnMOF-MNs reached 92.30 ± 2.32% hair coverage, which was significantly higher than that of the Model group (32.84 ± 13.40%), the BMN group (35.03 ± 16.62%), and the control group (86.29 ± 1.95%) (Figure 6A-C). In general, MN showed significant therapeutic effect in improving hair regrowth by reversing the adverse effects of DHT on DPCs and by providing a favorable microenvironment in AGA animal models.

## Conclusion

In summary, we fabricated multifunctional ZnMOF-MNs that promoted hair growth by reversing the hostile microenvironment under hair loss conditions. ZnMOF-MN is a painless and bloodless method with sufficient mechanical strength to penetrate the stratum corneum and creates temporary open channels for the transdermal delivery of ZnMOF. Zn ions and curcumin released from ZnMOF exerted a strong effect on ROS damage and cell apoptosis, whereas curcumin also potently inhibited the adverse effects of DHT. Taken together, these provide a favorable microenvironment for hair regrowth *in vivo* and *in vitro*. More importantly, compared with the traditional method of oral administration, the topical release of zinc and curcumin through ZnMOF-MNs can reduce the risk of chronic toxicity and the eliminating effect. Thus, ZnMOF-MN was proved to be an effective, biosafe, and comprehensive solution for promoting hair growth under various conditions.

## Methods

### Chemicals and Materials

γ-PGA (MW = 1000-15000) was purchased from Sai Taisi Biological Technology Co., Ltd. (China). Curcumin (B20614, HPLC≥98%) was purchased from Shanghai Yuanye Bio-Technology Co., Ltd. (China). DHT was purchased from Beijing Solarbio Science & Technology Co., Ltd. (China). The MN patch molds purchased from Taizhou Weixin Technology Co., Ltd. (China), was composed of a 10 × 10 array and produced 260 μm × 260 μm × 500 μm (W × L × H) MN tips. Dulbecco's Modified Eagle Medium/Nutrient Mixture F-12 (DMEM/F-12) cell culture medium and fetal bovine serum (FBS) were purchased from Gibco (UK).

### Cell Culture

C57BL/6-derived primary DPCs were purchased from iCell Bioscience Inc. (China). Cells were cultured in DMEM/F-12 medium supplemented with 10% FBS and 1% penicillin-streptomycin, and incubated in a humidified chamber containing 5% CO_2_ at 37 °C. Cells used in this study were all passage 3.

### Fabrication of ZnMOF and ZnMOF Hydrogel

ZnMOF was synthesized according to a previously reported method 32. At the beginning, 100 mg ZnSO_4_·7H_2_O was resolved in 10 mL ddH_2_O, while 120 mg curcumin was first resolved in 10 mL ddH_2_O under the pH 12 (adjusted with 10 mol L^-1^ KOH). Then, they were mixed and stirred thoroughly. The mixture was heated at 50 °C for 24 h in a muffle furnace (Sxi-8-10, Xinhan Instrument Equipment Co., Ltd. (China). The orange-yellow solid was obtained via separation in a centrifuge (12000 rpm, 15 min, 4 °C) and washed with 95% ethanol and ddH_2_O each for twice. To produce the ZnMOF hydrogel, 20 mg ZnMOF solid was first dissolved in 1 mL DMSO and then diluted to 1 mg mL^-1^ with ddH_2_O. Then, 600 μL diluted ZnMOF solution was added to 350 mg γ-PGA. The mixture was then sonicated for 30 min at room temperature using an ultrasonic cleaner. Bubbles were removed via centrifugation (12000 rpm, 15 min, 4 °C). For the hydrogel used to produce BMN, the diluted ZnMOF solution was replaced with ddH_2_O. The ZnMOF solution and ZnMOF-MN extract were neutral (evaluated by pH indicator strips), as shown in Figure S9.

### Fabrication and Characterization of MN patch

To produce ZnMOF-MNs, the microcavities of the needle tips were filled completely with ZnMOF hydrogel and centrifuged for 5 min (3000 rpm, 5 min, room temperature). A cotton swab was used to wipe off the extra gel. The filled mold was placed overnight in a 40 °C oven to dry.

### Hygroscopicity Test

The dried MN patches were placed in a sealed tank at 75% humidity. Photographs of the tips were captured every 5 min with a Scanning Electron Microscope (JCM-5000, NeoScope, USA).

### ZnMOF Release Profile

To determine the ZnMOF release profile, two patches of ZnMOF-MN were immersed in 2 mL PBS. The absorbance of the supernatant (extract) at 430 nm was determined by using a spectrometer (Thermo Fisher Scientific, Waltham, MA, USA) at 5 min, 10 min, 20 min, 30 min, 1 h, 2 h, 4 h, 8 h, 18 h, and 24 h. Subsequently, the corresponding concentration of ZnMOF in the supernatant was calculated from the absorbance using the calibration curve extracted shown in Figure 1I. The release ratio was calculated by the following equation:



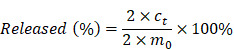



where 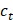 refers to the calculated concentration of ZnMOF at 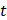 time, and 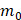 refers to the mass of the ZnMOF loaded to the MN patch. 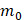
was calculated by the following equation:



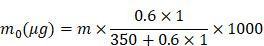



where 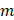 refers to the total weight of the two dried ZnMOF-MN patches.

To prepare the ZnMOF-MN extract for *in vitro* studies, 40 mL DMEM/F-12 culture medium was used, instead of PBS. Considering that the releasing percentage of Zn-MOF was 57.86% at 2 h, and the weight of every two ZnMOF patches was 257.8 ± 7.59 mg, the concentration of ZnMOF in the supernatant harvest at 2 h was estimated to be 6.46 μg mL^-1^. For the BMN extract, ZnMOF patches were replaced with BMN patches.

### Skin Penetration Study

C57BL/6 mice were anesthetized with isoflurane, and their backs were shaved. Then, a ZnMOF-MN patch was rapidly pressed into the depilated skin and held for 1 min (Figure S10). After removing the patch, images of the recovery procedure were recorded every 5 min before the holes faded. The skin was then peeled off and embedded vertically in an optimal cutting temperature compound (OCT). Transverse cryosections (8-μm-thick) were cut off and placed on glass slides for examination with a fluorescence microscope (Leica DM 2500, Germany).

### *In Vitro* Cytotoxicity Test

The CCK-8 kit (Dojindo Laboratories, Japan) was used to evaluate cell viability. Every 3500 DPCs were seeded into one well of a 96-well plate overnight; then, the medium was replaced with culture medium without ZnMOF or with various concentrations (0, 2.5, 5, 7.5, and 10 μg mL^-1^) of ZnMOF for 48 h. Each group was replicated six times. CCK-8-DMEM/F-12 solution (1:9) was used for incubation at 37 °C. After 2 h, the absorbance at 450 nm was evaluated by a spectrometer (Thermo Fisher Scientific, Waltham, MA, USA), and the results were then statistically analyzed and plotted. DPCs cultured in DMEM/F-12 with 0.1% DMSO were used as controls.

### *In Vitro* Antioxidant Tests

The viability of DPCs after treatment with different antioxidant treatments was determined by CCK-8 kit, in the same manner as described previously. For the experimental groups, cells were treated with 5 μg mL^-1^ ZnMOF solution, BMN extract, or ZnMOF-MN extract (approximately containing 6.46 μg mL^-1^ ZnMOF) for 24 h and then incubated with 3 mM H_2_O_2_ for 30 min. DPCs cultured in DMEM/F-12 with 0.1% DMSO were used as controls.

### *In Vitro* Zinc Deficiency Test

The viability of DPCs was determined by CCK-8 kit, in the same manner as described previously. TPEN (N, N, N', N'-Tetrakis (2-pyridylmethyl) ethylenediamine) of 5 μM was used to mimic the zinc deficient microenvironment. For the experimental groups, cells were treated with 5 μg mL^-1^ ZnMOF solution, BMN extract, or ZnMOF-MN extract (approximately containing 6.46 μg mL^-1^ ZnMOF) for 24 h. DPCs cultured in DMEM/F-12 with 0.1% DMSO were applied as the control group.

### *In Vitro* Dead Cell Staining

Propidium iodide (PI; Dojindo Laboratories, Japan) was used to stain dead cells. Every 20,000 DPCs were seeded in one well of a 24-well plate for overnight, then the medium was replaced with 5 μM TPEN. For the experimental groups, cells were treated with 5 μg mL^-1^ ZnMOF solution, BMN extract, or ZnMOF-MN extract (containing approximately 6.46 μg mL^-1^ ZnMOF) for 24 h. Each group was triplicated. DPCs cultured in DMEM/F-12 with 0.1% DMSO were used as the control group. Dead cells, stained red with PI, were imaged by a fluorescence microscope (Leica DM 2500, Germany). The number of dead cells was automatically counted under a high lens by Image J 1.53c (National Institutes of Health, USA).

### *In Vitro* Apoptosis Assay

The Annexin V-FITC assay (556547, BD, USA) was used to evaluate cell apoptosis. DPCs were incubated with 5 μM TPEN for 24 h. Then 5 μg mL^-1^ ZnMOF solution, BMN extract, or ZnMOF-MN extract were added in corresponding groups. DPCs cultured in DMEM/F-12 (0.1% DMSO) without TPEN were used as the control group. After 24 h of treatment, cells were collected by using Trypsin (0.25%) and washed twice with PBS. Next, 5 μL Annexin V-FITC, 5 μL of PI and 500 μL of binding buffer were added to each group and gently mixed up. After 15 min of incubation, apoptosis was detected with a flow cytometer (Beckman Coulter, USA). The sum of the cell proportion in upper right (UR) and lower right (LR) represents the total apoptosis rate.

### RNA Isolation and RT-qPCR

10 0000 cells per well were seeded in a 6-well plate. DPCs cultured in DMEM/F-12 with 0.1% DMSO were applied as the control group. For the experimental groups, cells were incubated with 100 μM DHT, and then treated with or without 20 μM curcumin, 2.5 μg mL^-1^ ZnMOF solution, BMN extract, or diluted ZnMOF-MN extract (containing approximately 3.23 μg mL^-1^ ZnMOF) for 24 h. Then, the cultured DPCs were harvested for total RNA extraction using a RNA purification kit (C10310, EZBioscience Co., Ltd., USA) according to the manufacturer's instruction. cDNA was synthesized from 1 µg total RNA per sample using 4×Ezscript RT Mix II ((B0004DP, EZBioscience). The cDNA was amplified using RT-qPCR in a real-time thermal cycler (Stratagene, La Jolla, CA, USA) with 2×STBR Green qPCR Mix (ROX2 Plus) (A0001-R2, EZBioscience). The housekeeping gene GAPDH was used as internal control. Each assay was performed in triplicates.The sequences of mouse primers (Sangon Biotech (Shanghai) Co., Ltd., China) for RT-qPCR analysis are shown in Table S1.

### *In Vivo* Wound Healing Evaluation

In this study, male C57/BL6 mice (6-8 weeks old) were used to establish a wound-healing model. All animal study protocols were approved by the Institutional Animal Care and Experiment Committee of Shanghai Jiaotong University School of Medicine. Fifteen mice were randomly divided into three groups (control, BMN, and ZnMOF-MN group). Surgery was performed on the back of each mouse (n = 3) to create a circular wound with a diameter of approximately 9 mm (the inner diameter of the circular peeler is 10 mm). An air isoflurane anesthesia machine was used to anesthetize the mice for a short time and establish a wound operation with total skin loss. The ZnMOF-MN patches were degraded within 30 min (Figure S11). The BMN and ZnMOF-MN groups were treated only once with an MN patch on day 0 after the wound model was established. Each mouse was reared in a single cage with sufficient water and food. The mice in each group were photographed at 0, 3, 5, 7, 9, 11, 13, 15, and 17 days after the wound treatment, and the wound area was calculated using the ImageJ 1.53c software (National Institutes of Health). The wound area rate was calculated using the following formula:



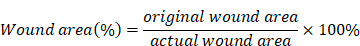



### Hair Growth Ratio Evaluation

Male C57BL/6 mice (6-8 weeks old) were used to establish an androgenic alopecia model. All animal study protocols were approved by the Institutional Animal Care and Experiment Committee of Shanghai Jiaotong University School of Medicine. Twenty mice were randomly divided into 4 groups (control, Model, BMN, and ZnMOF-MN groups; n = 5) (Figure 6A and Figure S8). DHT (2 mg kg^-1^) was subcutaneously injected to mice in the model group, BMN group, and ZnMOF-MN group, and equal volume of normal saline was injected into mice in the control group once daily for two weeks. Afterwards, an area of 4 cm^-2^ (2 cm × 2 cm) from the dorsal portion of the mice in the telogen phase was gently shaved using with an electric hair clipper and depilated with hair removing cream (Day 1). BMNs or ZnMOF-MNs were applied in the middle of the depilated area on day 1, 4, 7, and 10. The MN patch was pressed for 1 min and 4 patches were used consecutively. Digital images of the skin and hair were obtained.

### Histology and Immunohistochemistry Staining

On day 17, mice of wound healing models were sacrificed. Taking the remaining wound as the center, round skin of 6 mm diameter was removed as the sample. Soaked in 4% paraformaldehyde at 24 h, samples were embedded in wax blocks and then prepared into 5-μm-thick sections for histological staining. The number of hair follicles was determined by using H & E staining. Detection of CD31+ and Ki67+ cells among tissues. The tissue sample sections were placed in rabbit monoclonal anti-CD31 antibodies (ab182981, Abcam, 1:400) or rabbit monoclonal anti-Ki67 antibodies (ab15580, Abcam, 1:200), and incubated overnight at 4 °C. The sections were then incubated with HRP-labeled, anti-rabbit antibodies at 37 °C. A 3,3′-diaminobenzidine substrate kit (Boster, Wuhan, China) was used for staining. The ImageJ 1.53c software (National Institutes of Health, USA) was used to calculate hair follicle density, capillary density, and the ratio of Ki67+ cells in the wound samples.

### Immunofluorescence staining

On day 18, mice of androgenic alopecia models were sacrificed. An area of 1 cm^-2^ (1 × 1 cm) from the center of the shaved area was removed as the sample. Soaked in 4% paraformaldehyde for 24 h, the samples were embedded in wax blocks and then prepared into 5-μm-thick sections for immunofluorescence staining. The tissue sample sections were permeabilized using 0.1% Triton X-100 (Sigma-Aldrich) and blocked with 5% goat serum in PBS, and incubated overnight with rabbit monoclonal anti-CD31 antibodies (ab305267, Abcam, 1:50) or rabbit monoclonal anti-Ki67 antibodies (ab15580, Abcam, 1:200) at 4 °C. Finally, the samples were washed with PBS and counterstained with 4,6-diamidino-2-phenylindole (DAPI). ImageJ (National Institutes of Health, USA) was used to calculate hair follicle density, capillary density, and ratio of Ki67+ cells in the wound samples.

### Statistical analysis

All data were collected in triplicate and reported as mean and standard deviation. The comparison of two conditions was evaluated by the unpaired t-test. Except for the data of wound areas, angiogenesis and immunofluorescent intensity were analyzed with Image J, all data was evaluated with GraphPad. p<0.05 was considered statistically significant differences.

## Supplementary Material

Supplementary figures and table.

## Abbreviations

γ-PGA: gamma-polyglutamic acid
ZnMOF: curcumin-zinc framework
MOFs: metal-organic frameworks
MN: microneedle
ZnMOF-MN: curcumin-zinc framework encapsulated into gamma-polyglutamic acid microneedle patch
Zn: zinc
Zn^2+^: zinc ion
FE-SEM: field emission scanning electron microscope
DLS: dynamic light scattering
XRD: X-ray diffraction
DPCs: mouse dermal papilla cells
AGA: androgenic alopecia
ROS: reactive oxygen species
DHT: dihydrotestosterone
