# Supplementary material for: Curcumin-zinc framework encapsulated microneedle patch for promoting hair growth
Source: Theranostics. 2023 Jun 26;13(11):3675–88. doi: 10.7150/thno.84118 (PMC10334826; doi:10.7150/thno.84118)
Supplement: Supplementary file 1 — Supplementary figures and table. [file thnov13p3675s1.pdf]

Supplementary Information

Curcumin-zinc framework encapsulated microneedle patch for promoting hair growth

Yating Yang[1, #], Pei Wang[1, #], Yan Gong[1], Ziyou Yu[1,*], Yuci Gan[1], Peizhe Li[1], Wei Liu[1,*] and Xiansong Wang[1,*]

[1] Department of Plastic and Reconstructive Surgery, Shanghai Key Laboratory of Tissue Engineering, Shanghai Ninth People's Hospital, Shanghai Jiao Tong University School of Medicine, Shanghai 200011, China.

[#] These authors contributed equally to this work

[*] Corresponding author.

E-mail address: wonderluis@sjtu.edu.cn (X. Wang), liuwei_2000@yahoo.com (W. Liu), yuziyou2013@126.com (Z. Yu)

Table S1. Sequences of primers for RT-qPCR analysis

Gene	Sequence (5' to 3')
β-Actin	*F: GGCTGTATTCCCCTCCATCG*
	R: CCAGTTGGTAACAATGCCATGT
DKK1	*F: CTCATCAATTCCAACGCGATCA*
	R: GCCCTCATAGAGAACTCCCG
CCND1	*F: GCGTACCCTGACACCAATCTC*
	R: CTCCTCTTCGCACTTCTGCTC
AMER3	*F: TAGCGTGCAGATTTCTCACGA*
	R: GAACAGGGACTTGTTTGGGAG
LEF1	*F: TGTTTATCCCATCACGGGTGG*
	R: CATGGAAGTGTCGCCTGACAG
HSD17B2	*F: ATGAGCCCGTTTGCCTCTG*
	R: CCACAGGTAACAAGTCTTGGTC
SRD5A1	*F: GAGTTGGATGAGTTGCGCCTA*
	R: GGACCACTGCGAGGAGTAG
Caspase-3	*F: ATGGAGAACAACAAAACCTCAGT*
	R: TTGCTCCCATGTATGGTCTTTAC
Caspase-9	*F: TCCTGGTACATCGAGACCTTG*
	R: AAGTCCCTTTCGCAGAAACAG
PCNA	*F: TTTGAGGCACGCCTGATCC*
	R: GGAGACGTGAGACGAGTCCAT

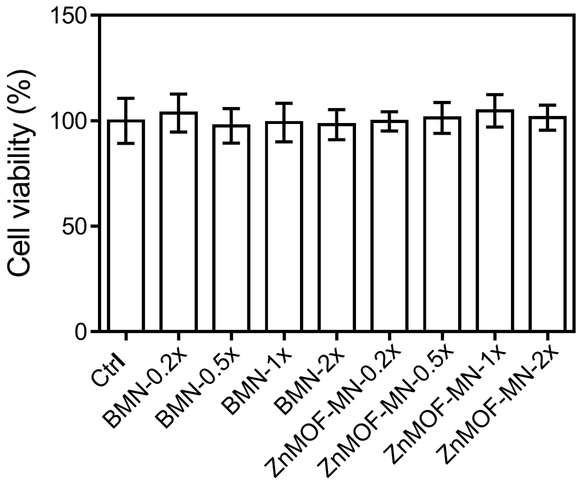

Figure S1. Cell viability of DPCs after BMN extract or ZnMOF-MN extract treatment for 48 hours. The concentration of extract where two pieces of BMN or ZnMOF-MN patches were immersed into a 40 mL culture medium was defined as 1 x. Ctrl represents the control group. No statistical significance was observed among all groups.

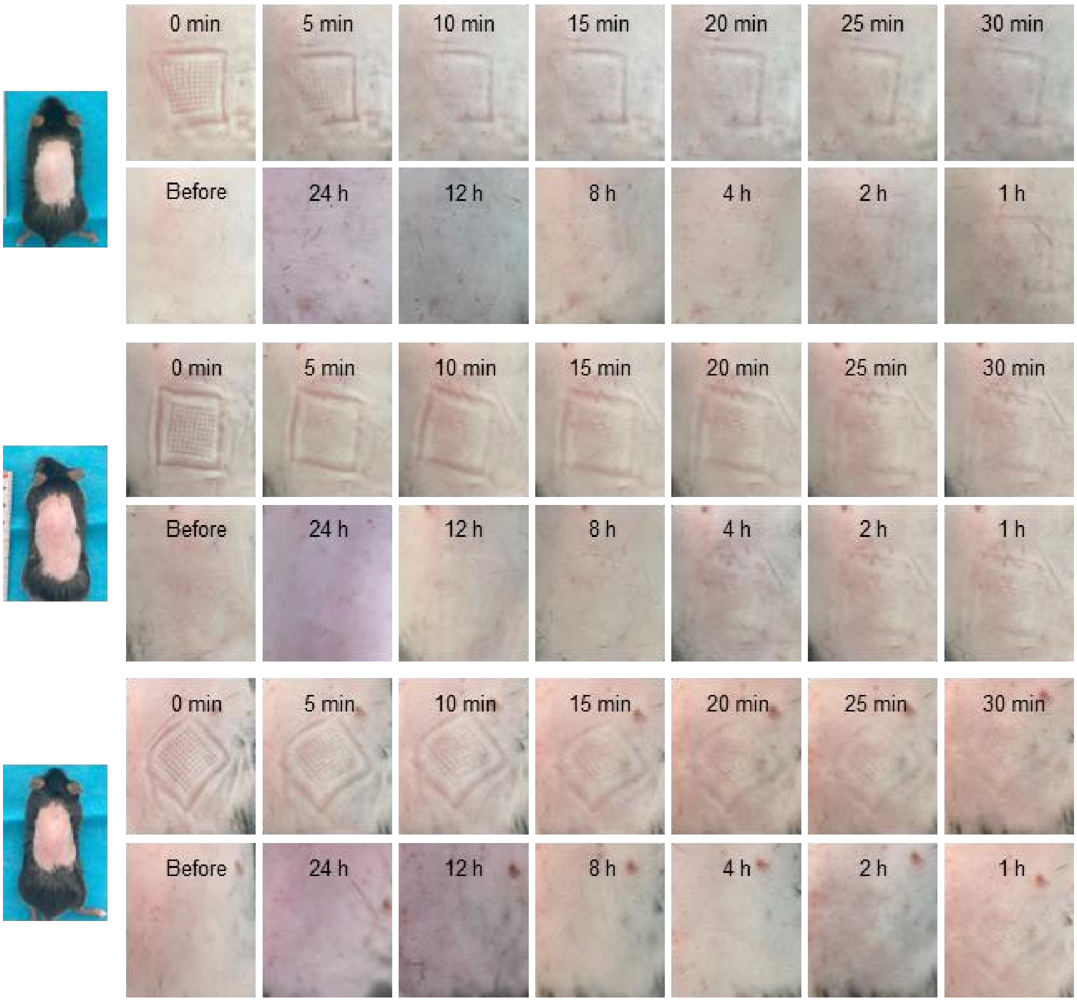

Figure S2. The recovery procedure after ZnMOF-MN penetration in the back skin of C57BL/6 mice. Temporary holes created by ZnMOF-MN penetration were recovered within 30 min without bleeding. No obvious penetration-derived skin damage or inflammation were observed in all animals in 24 h.

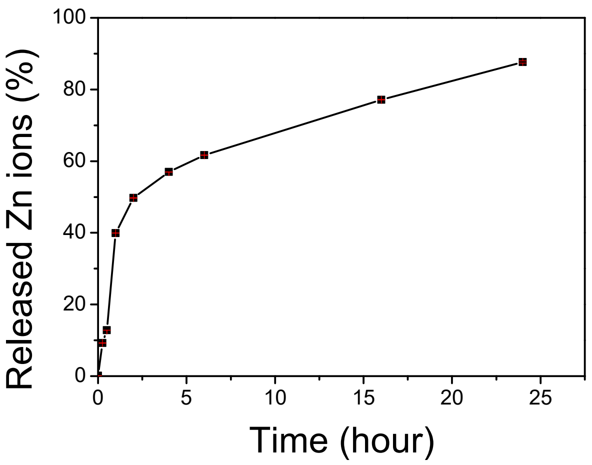

Figure S3. Release profile of Zn ions (Zn^{2+}) from two ZnMOF-MN patches in 2 mL PBS within 24 h. To determine the Zn release profile, two patches of ZnMOF-MN were immersed in 2 mL PBS. The supernatant (200 μL) was collected at 0 min, 15 min, 30 min, 1 h, 2 h, 4 h, 8 h, 14 h, and 24 h. The Zn^{2+} concentration was measured via Inductively coupled plasma-Mass Spectrometry (ICP-MS; Agilent 7800, USA). Error bars were presented in red.

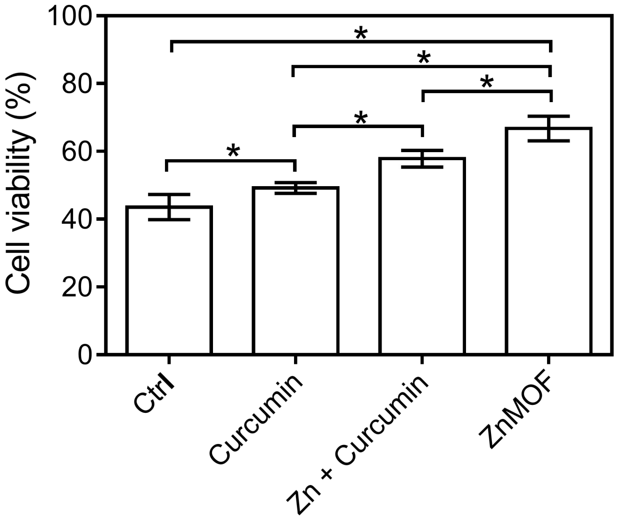

Figure S4. Cell viability of DPCs. DPCs were treated with curcumin solution (10 μM), Zn (5 μM) and curcumin (10 μM) solution, and ZnMOF solution (5 μg mL^{-1}) for 24 h and then incubated with 3 mM H_2O_2 for 30 min. * $p < 0.05$

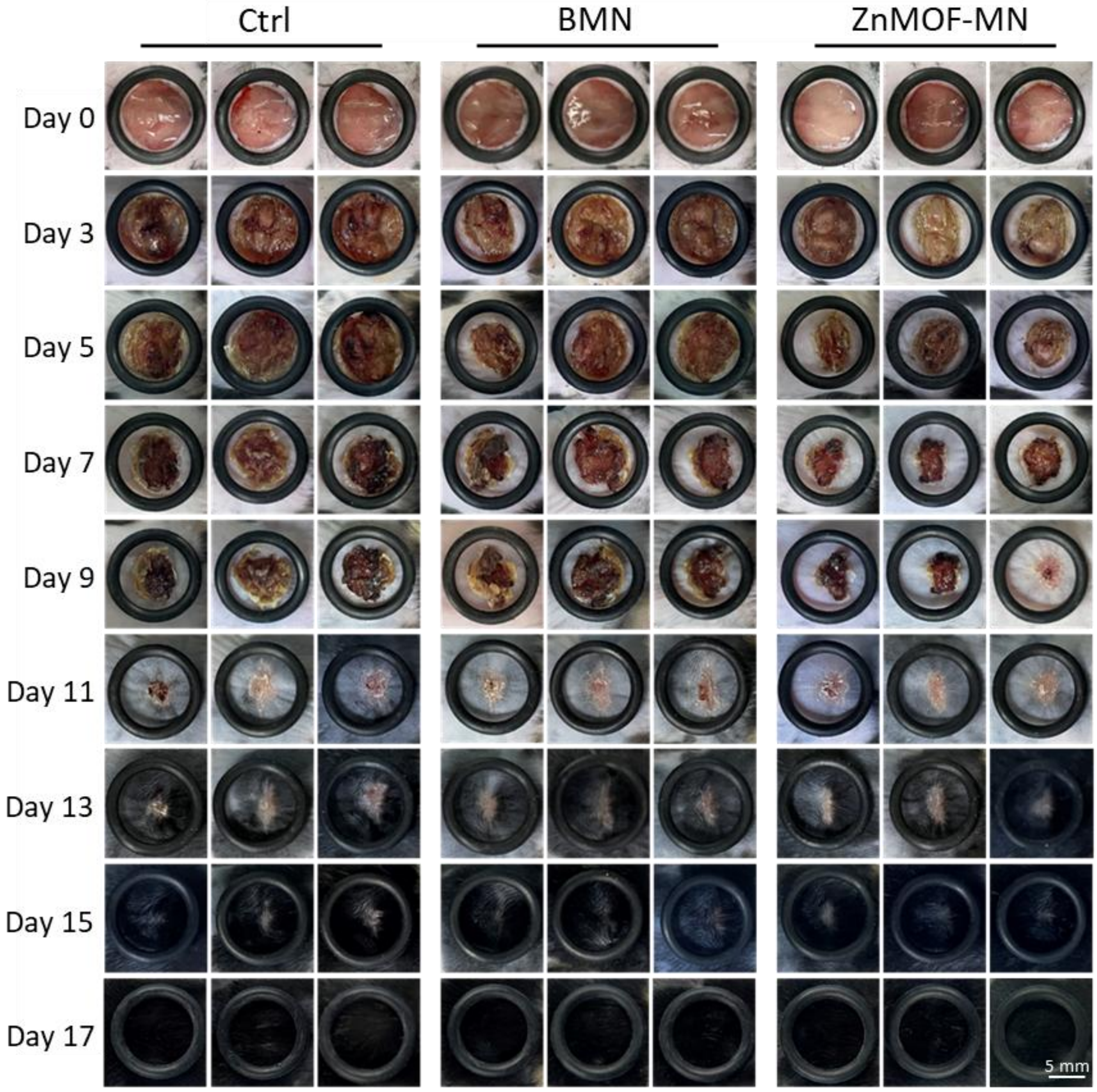

Figure S5. Images of the wound areas and hair growth conditions in wound healing models on day 0, 3, 5, 7, 9, 11, 13, 15, and 17, respectively.

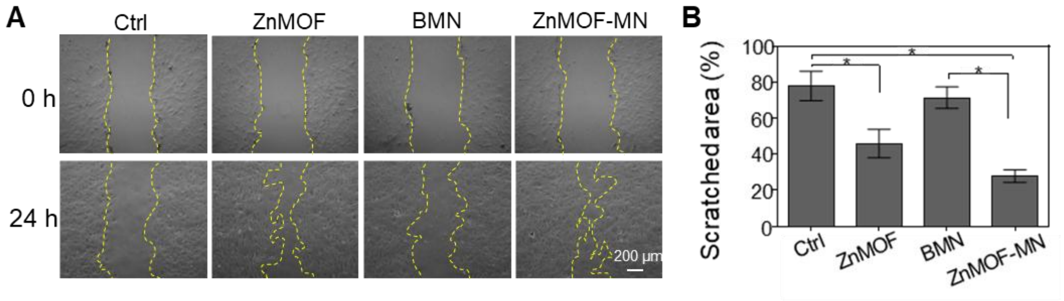

Figure S6. *In vitro* wound healing scratch assay. A. The scratch assay was used to evaluate the cell migration. The cell monolayer was scratched with a 1000 μL-pipette tip when DPCs reached more than 90% confluence. For the experimental groups, cells were then treated with 5 μg mL^{-1} ZnMOF, BMN extract, or ZnMOF-MN extract (approximately containing 6.46 μg mL^{-1} ZnMOF). DPCs cultured in DMEM/F-12 with 0.1% DMSO were applied as the control group (Ctrl). Images were captured at 0 h and 24 h after scratching. The percentage of scratched area was automatically measured by the Image J 1.53c (National Institutes of Health, USA). Representative images of the scratch assay treated with DMEM/F-12 containing 0.1% DMSO (Ctrl), 5 μg mL^{-1} ZnMOF, BMN extract, or ZnMOF-MN extract (approximately containing 6.46 μg mL^{-1} ZnMOF). After the scratches had been made, the images were taken immediately and 24 h after different treatments. The yellow dashed line indicates the borders of areas without migrating cells. **B.** Quantitative analysis of the scratched area (%) after different treatments. n = 3, *$p < 0.05$.

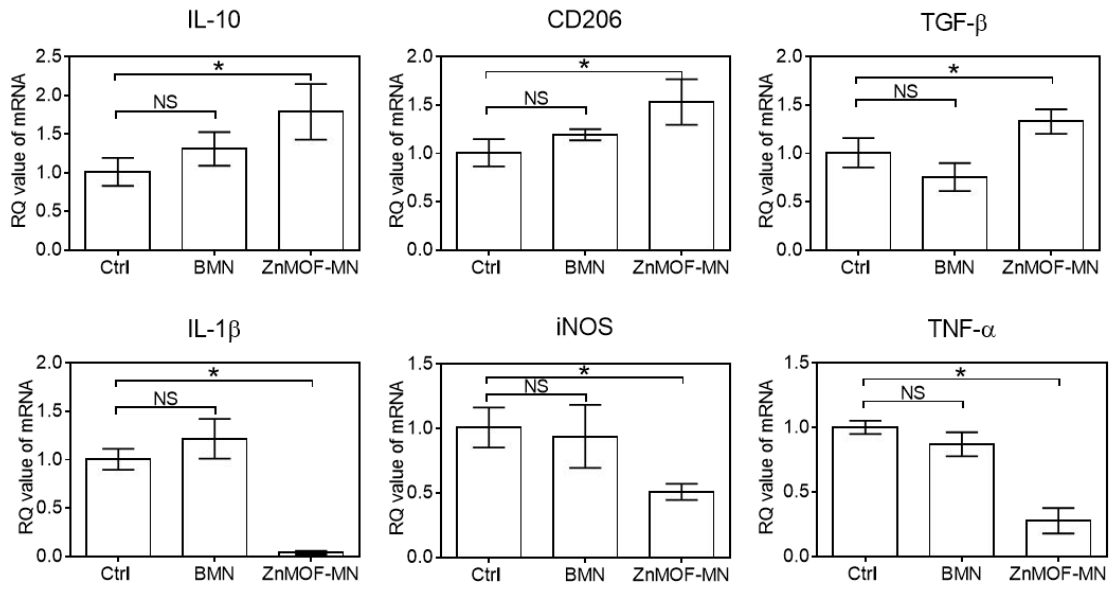

Figure S7. Gene expression of M1 and M2 phenotype markers in RAW264.7 macrophage cell lines (M0). RAW264.7 cell lines were donated by Shanghai Key Laboratory of Tissue Engineering. Cells were cultured in Roswell Park Memorial Institute (RPMI)-1640 medium supplemented with 10% FBS and 1% penicillin-streptomycin, and incubated in a humidified chamber containing 5% CO_2 at 37 °C. RNA Isolation and RT-qPCR procedures were consistent with the previous description. M2 markers, including IL-10, CD206, and TGF-β were significantly increased, while M1 markers, including IL-1β, iNOS, and TNF-α, were significantly decreased after 24 h ZnMOF-MN extract treatment. *$p < 0.05$; NS represents no statistical significance. Ctrl represents the control group (0.1% DMSO). The sequences of mouse primers (Sangon Biotech (Shanghai) Co., Ltd., China) for RT-qPCR analysis are shown in Table S1.

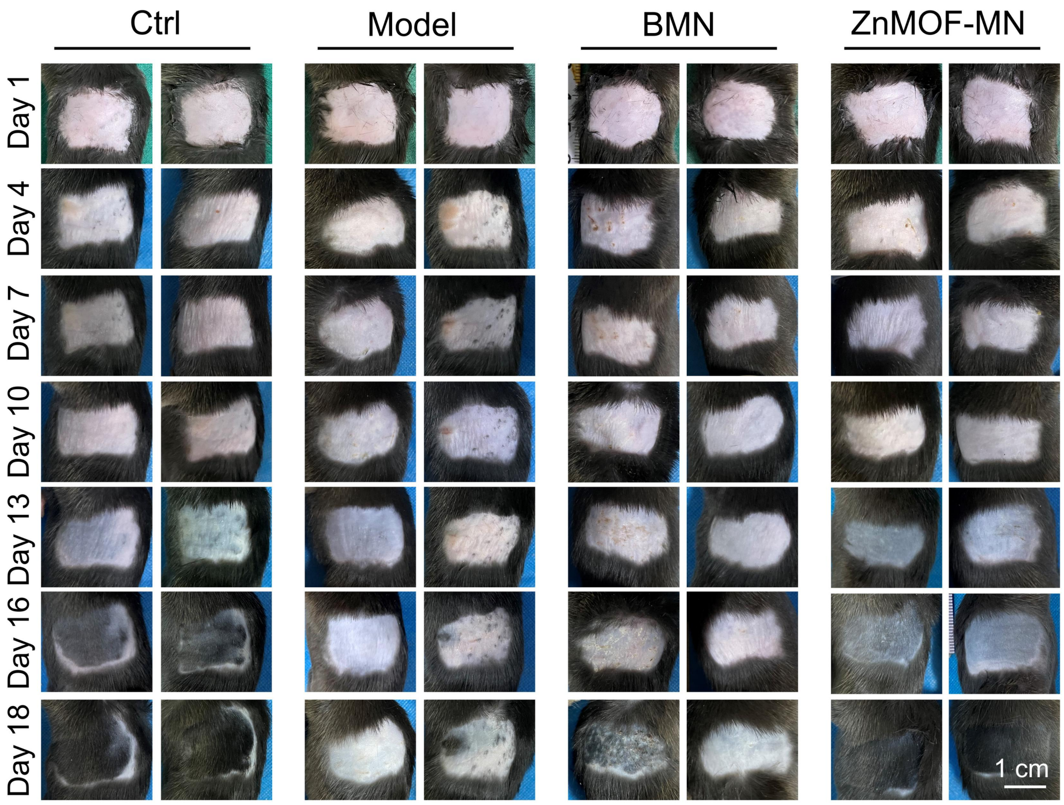

Figure S8. Photographic pictures of hair regrowth conditions in the rest two animals of each group on day 1, 4, 7, 10, 13, 16, and 18, respectively.

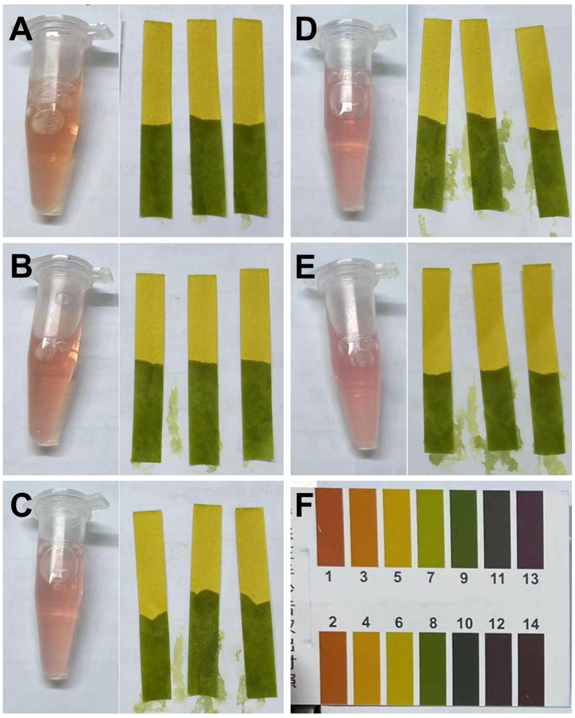

Figure S9. The pH values of ZnMOF solution or ZnMOF-MN extract evaluated by pH indicator strips. A. ZnMOF/F12 medium solution, 20 μg mL^{-1}. **B.** ZnMOF/F12 medium solution, 5 μg mL^{-1}. **C.** ZnMOF/F12 medium solution, 2.5 μg mL^{-1}. **D.** ZnMOF-MN extract, ~6.46 μg mL^{-1}. **E.** ZnMOF-MN extract, ~3.23 μg mL^{-1}. **F.** Reference color card.

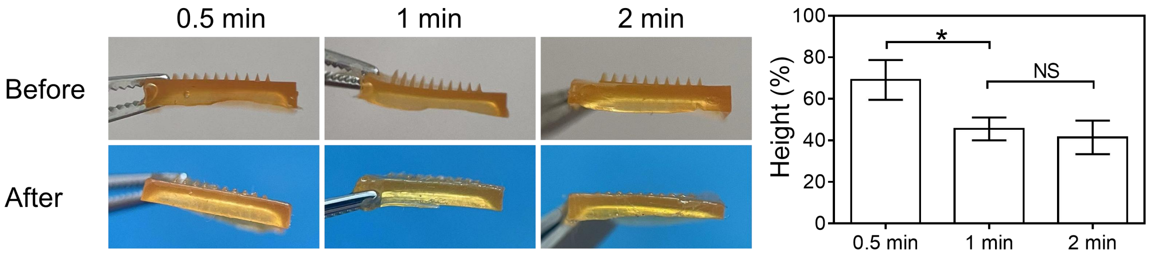

Figure S10. Images and analysis of height reduction in ZnMOF-MN tips after being pressed into the mouse skin. * $p < 0.05$, NS represents no statistical significance.

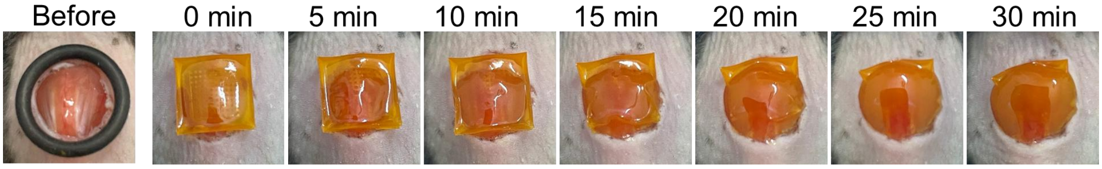

Figure S11. Images of *in vivo* ZnMOF-MN degradation test within 30 min.
